# The Necessity for a Higher Standard

**Published:** 1888-08

**Authors:** 


THE NECESSITY FOR A HIGHER STANDARD.

The question naturally arises as to how we shall meet the exigencies of our anomalous position in which we have been placed by the action of the American Medical Association. Recognition has been extended to us upon the standing of the few, and not upon the attainments of the entire profession. We have many liberally educated men in our ranks who possess only the one degree, and these men have, with those medically educated, stood sponsors, so to speak, for the whole profession. We ought not to plume ourselves too highly upon our sudden elevation to a prominent position, but should, in view of our many deficiencies, resolve to acquit ourselves of the confidence reposed in us. In order to do this it is absolutely essential that the college term be extended to three years. It is not a matter of degrees, but qualifications. We may educate every student so that when he receives his dental degree he shall be qualified and receive recognition as a specialist in medicine. The only way to accomplish the much needed extension of time required for graduation in dentistry is for the profession, through its societies and journals, to demand that the schools of the country extend the time and raise the standard for graduation.

That the profession is desirous of such an advance is evidenced by the resolutions that have been passed, the present summer, by the Illinois State Society, the Connecticut Valley and the Massachusetts Societies in union meeting—by the Maine State Society and the New Jersey State Society. The hearty reception given President Fundenberg's address before the Pennsylvania State Society showed the sentiment of that organization, and we have no doubt that if such a resolution had been presented that it would have been passed without any difficulty.

Previous to the passage of State laws looking towards the regulation of dental practice, our colleges were amenable to no power save themselves. The establishment of State Boards of Censors, in a certain measure, put a stop to the indiscriminate granting of degrees. It is true that a college may grant a degree, but such degree no longer carries with it the license to practice, unless the State Board of Censors in the State where the individual shall elect to locate shall see fit to honor the degree; otherwise, he must come before the Board and pass such examination as they may choose to prescribe for him. They have almost arbitrary power in the matter, consequently the colleges do well to consult the wishes of the National Association of Dental Examiners. They have more power than the National Association of Dental Faculties. The latter may suggest; the former, demand. The much needed extension of time will come with better grace if suggested by the colleges themselves, than if they are compelled to adopt it at the demand of the profession. The PRACTITIONER has determined to keep up the battle-cry in this direction, until some marked step in advance is obtained. We mean, if possible, to start the ball rolling and get a free expression on the subject. We cannot ignore the question, but must face it manfully. It is either advance or retreat. We occupy an anomalous position, and must decide one way or the other. Some will not admit these alternatives, but hold that we shall remain a separate profession. Such a position is, however, in the present state of affairs, logically untenable, for it would require the dismission of medically educated teachers from our dental faculties; and that would surely be retrogression. No, there is only one way, and that is to advance—advance, for such is the spirit of the age.

The demand for higher education is met by the statement from the faculties that they cannot make "bricks without straw." Give us better material, say they, and we shall turn out better men. The rejection of unqualified men lies with the schools themselves. Many a good dentist is spoiled in the making—the requirements are too light—honors too easy. Students, as a rule, do work only sufficient to obtain their degree; but do the work they will, no matter what it is. They must meet the requirements in order to obtain the right to practice dentistry and a sufficient number will meet the demand even if they have to study three years. Those who will not are the very ones we can best afford to lose from the ranks. We want ambitious men, and none others. Dentistry pays those who follow it as a profession sufficiently well to recompense the best educated men to take it up as a life work. It pays much better, considering the class of talent engaged in its practice, than does law or medicine. Once place the requirements for matriculation and graduation on the same plane as the other professions, and a different class of men will necessarily engage in the ranks. It is just as honorable a calling as medicine, and more so than law; and the only reason it is not so considered is because of the fact that comparatively uneducated men may enter its ranks. The spirit of unrest that pervades the profession to-day bespeaks an advance all along the line. The public demand it of us, because they compensate us fully for our services.

Let us therefore raise the standard and purge our ranks of incompetent men.

